# Ancestry as a potential modifier of gene expression in breast tumors from Colombian women

**DOI:** 10.1371/journal.pone.0183179

**Published:** 2017-08-23

**Authors:** Silvia J. Serrano-Gómez, María Carolina Sanabria-Salas, Jone Garay, Melody C. Baddoo, Gustavo Hernández-Suarez, Juan Carlos Mejía, Oscar García, Lucio Miele, Laura Fejerman, Jovanny Zabaleta

**Affiliations:** 1 Grupo de investigación en Biología del Cáncer, Instituto Nacional de Cancerología, Bogotá D. C, Colombia; 2 Programa de doctorado en Ciencias Biológicas, Pontificia Universidad Javeriana, Bogotá D. C, Colombia; 3 Stanley S. Scott Cancer Center, LSUHSC, New Orleans, LA, United States of America; 4 Tulane University School of Medicine, New Orleans, LA, United States of America; 5 Grupo de Patología, Instituto Nacional de Cancerología, Bogotá D. C, Colombia; 6 Grupo de Seno y Tejidos blandos, Instituto Nacional de Cancerología, Bogotá D. C, Colombia; 7 Department of Genetics, LSUHSC, New Orleans, LA, United States of America; 8 Department of Medicine, Institute of Human Genetics, University of California San Francisco, San Francisco, CA, United States of America; 9 Department of Pediatrics, LSUHSC, New Orleans, United States of America; Ohio State University Wexner Medical Center, UNITED STATES

## Abstract

**Background:**

Hispanic/Latino populations are a genetically admixed and heterogeneous group, with variable fractions of European, Indigenous American and African ancestries. The molecular profile of breast cancer has been widely described in non-Hispanic Whites but equivalent knowledge is lacking in Hispanic/Latinas. We have previously reported that the most prevalent breast cancer intrinsic subtype in Colombian women was Luminal B as defined by St. Gallen 2013 criteria. In this study we explored ancestry-associated differences in molecular profiles of Luminal B tumors among these highly admixed women.

**Methods:**

We performed whole-transcriptome RNA-seq analysis in 42 Luminal tumors (21 Luminal A and 21 Luminal B) from Colombian women. Genetic ancestry was estimated from a panel of 80 ancestry-informative markers (AIM). We categorized patients according to Luminal subtype and to the proportion of European and Indigenous American ancestry and performed differential expression analysis comparing Luminal B against Luminal A tumors according to the assigned ancestry groups.

**Results:**

We found 5 genes potentially modulated by genetic ancestry: *ERBB2 (*log2FC = 2.367, padj<0.01), *GRB7 (*log2FC = 2.327, padj<0.01), *GSDMB (*log2FC = 1.723, padj<0.01, *MIEN1 (*log2FC = 2.195, padj<0.01 and *ONECUT2 (*log2FC = 2.204, padj<0.01). In the replication set we found a statistical significant association between *ERBB2* expression with Indigenous American ancestry (p = 0.02, B = 3.11). This association was not biased by the distribution of HER2+ tumors among the groups analyzed.

**Conclusions:**

Our results suggest that genetic ancestry in Hispanic/Latina women might modify *ERBB2* gene expression in Luminal tumors. Further analyses are needed to confirm these findings and explore their prognostic value.

## Introduction

Breast cancer is a complex-multifactorial disease, consisting of a highly heterogeneous group of tumors with particular molecular features, prognosis and responses to therapy [1–4]. The first gene expression-based classification of breast cancer into intrinsic subtypes was published in 2000 [5] and identified estrogen receptor (ER) positive (ER+) subtypes Luminal A and B, and ER negative (ER-) subtypes basal-like and human epidermal growth factor receptor 2-enriched (HER2-enriched) [1, 6]. Subsequent studies showed differences in the outcomes according to intrinsic subtypes [7, 8]. Based on this classification, the best outcomes are observed for Luminal A tumors while basal-like and HER2-enriched are associated with worse outcomes.

The Luminal B subtype represents 30%– 40% of breast cancers [9, 10]. Despite expressing ERα and being amenable to endocrine therapy, they tend to be clinically more aggressive and have worse prognosis compared to Luminal A tumors. For example, it has been observed that survival curves for Luminal B tumors are similar to those from basal-like tumors after 10 years of follow-up [9]. Moreover, Luminal B tumors have higher risk of *de novo* resistance to endocrine therapies [7, 11] and at the molecular level, they are characterized by increased expression of cell proliferation genes or cell cycle regulators such as *MKI67* and *AURKA* [12–14]. Luminal B tumors more frequently receive high recurrence scores based on the Oncotype Dx gene expression signature and are more likely to benefit from cytotoxic chemotherapy, reaching higher percentages of pathologic complete response (pCR) when compared to Luminal A tumors [13, 15]. However, results on this issue have not been consistent [16–18].

Although there is still controversy as to whether Luminal A and Luminal B represent two different biological entities or a single entity that changes from one status to another through the acquisition of mutations [19–21], it is well established that this group of tumors are characterized by the expression of estrogen receptor α (*ESR1* gene), progesterone receptor (*PGR* gene) and cytokeratins characteristic of luminal cells such as cytokeratin 8 and cytokeratin 18 (*KRT8*, *KRT18*) [19]. Luminal A tumors are usually low-grade malignancies that show gains in 1q and loss in 16q. Luminal B tumors have a more complex profile of copy number variations (CNV). Amplifications at 8p11 (*FGFR1* locus), 8q21, 11q13, 17q12 (*ERBB2* locus) and 20q13 have been reported [13, 22, 23]. Based on gene expression profiles, these two subtypes share molecular patterns such as the expression of the *ESR1* gene and other genes such as *FOXA1* and *BCL2*, but their main difference is the high expression of proliferation genes such as *MKI67*, the survivin gene *BIRC5*, and the cyclin B1 (*CCNB1*) gene that characterizes the Luminal B subtype [14, 24, 25].

Hispanic/Latinas is a heterogeneous group with variable proportions of European, Indigenous American (IA) and African ancestries [26]. The Colombian population is one of the most diverse of Latin America [27]. Our group has previously reported that Luminal B, as defined by St. Gallen surrogate immunohistochemistry criteria, is the most common breast cancer intrinsic subtype among Colombian women [28]. The objective of the present work was to explore the molecular profile of Luminal tumors in Colombian women to assess the association between genetic ancestry and gene expression. We performed RNA-seq analysis in 42 formalin-fixed paraffin embedded (FFPE) tumor blocks previously classified as Luminal subtypes (21 Luminal A and 21 Luminal B) and with known genetic ancestry proportions [28]. We found 5 candidate genes (*ERBB2*, *GRB7*, *GSDMB*, *MIEN1* and *ONECUT2*) potentially modulated by genetic ancestry in Colombian-Latina patients with Luminal tumors.

## Materials and methods

### Patient selection

Patient’s sample blocks were selected from a database of 252 breast cancer patients with known genetic ancestry and with FFPE tumor specimens available. These 252 patients are part of a database of 301 breast cancer patients from Colombia that we have previously described [28]. For deep sequencing analysis, we selected 59 samples from the Andean region according to breast cancer intrinsic subtype and to the predominance of the European or IA ancestral fraction. Intrinsic subtypes were assessed using a panel of 6 immunohistochemistry (IHC) markers that included ER, progesterone receptor (PgR), the human epidermal growth factor receptor 2 (HER2), Ki-67, the Epidermal Growth Factor Receptor (EGFR), and Cytokeratin 5/6 (CK5/6), following the recommendations of St. Gallen 2013 consensus [29]. This study was approved by the Colombian National Cancer Institute ethics committee. Since we worked with de-identified FFPE tissues collected more than 3 years before the analysis done for this work, the Colombian NCI according to the Colombian laws, considered that no informed consent was required.

### Ancestry estimation

DNA was extracted from normal FFPE tissues using the RecoverAll™ Total Nucleic Acid Isolation Kit (Life Technologies, Carlsbad, CA) following the manufacturer’s recommendations. A panel of 106 Single Nucleotide Polymorphisms (SNPs) previously validated as Ancestry-Informative Markers (AIMs) was used to estimate individual genetic ancestry [30]. Genotyping was performed at the University of Minnesota Genomics Center using Sequenom technology. SNPs with call rate <90% or that deviated from Hardy-Weinberg equilibrium were removed from the analysis, leaving 80 SNPs for ancestry estimation. The software STRUCTURE version 2.222 [31] was used under an admixture model fixing the number of ancestral components to k = 3 to estimate Indigenous American (IA), European and African proportions for each of the samples. We used a burn-in period of 10,000 iterations followed by 50,000 additional iterations. Parental populations that include 42 Europeans (Coriell’s North American Caucasian panel), 37 West Africans (non-admixed Africans living in London, United Kingdom and South Carolina) and 30 Indigenous Americans (15 Mayans and 15 Nahuas) [30] were included to perform a supervised analysis of our samples.

### RNA-Seq sample preparation and data analysis

Hematoxylin and eosin-stained slides were evaluated by a pathologist to estimate the percentage of tumor present in the paraffin block selected. For cases with or more than 60% of tumor content, five 10μm sections were used for RNA extraction. For cases with less than 60% of tumor content, areas that contained tumor were marked to obtain 5 tumor cores using a 1-mm punch needle. RNA extraction was done using the RecoverAll™ Total Nucleic Acid Isolation Kit (Life Technologies, Carlsbad, CA) following the manufacturer’s recommendations. RNA-seq analysis was performed at the Stanley S. Scott Cancer Center’s Translational Genomics Core at LSUHSC. RNA was quantified by NanoDrop ND1000 Spectrophotometer (Thermo Scientific, Wilmington, USA) and its quality assessed with RNA 6000 Nano kit in the Agilent 2100 Bioanalyzer (Agilent Technologies, Santa Clara, CA). Even though the samples presented some RNA degradation, they were suitable for library preparation, based on the protocols and recommendations from Illumina.

Library preparation was performed in 59 samples from 1μg of total RNA using the TruSeq Stranded Total RNA Sample Preparation Kit (Illumina Inc., San Diego, CA). Briefly, isolated RNA was depleted of ribosomal RNA using the rRNA Removal Mix provided by the kit. Random hexamers were used for cDNA synthesis. Subsequently, cDNA was subjected to end repair, adapter ligation and size selection using AMPure XP beads (Beckman Coulter Inc., Brea, CA). Fragmentation step was omitted due to the sample quality, as recommended by the protocol. Libraries were quantified by Qubit dsDNA HS Assay Kit (Life Technologies, Carlsbad, CA) and the validation of the library size was performed in an Agilent Bioanalyzer using a DNA 1000 kit (Agilent Technologies, Santa Clara, CA) to verify the presence of a 260 base pair fragment. From the luminal tumors selected for library preparation, 42 (21 Luminal A and 21 Luminal B) had the expected size to proceed to the sequencing.

Sequencing was performed in a Genome Analyzer IIX (Illumina Inc) in a single-read 60 + 7 run (sequence plus index). For data analysis, FASTQ files were generated using CASAVA v1.8.1. FastQC software (Version 0.9.6) was used to evaluate the quality of the files. The trimming of adapter sequences from the reads was performed using fastq-mcf utility [32] and RSEM [33] was used to map single-end reads to reference transcriptome hg38 (Ensembl) and to provide read counts and normalized expression values for each case analyzed. The data analyzed in this publication have been deposited in NCBI’s Gene Expression Omnibus and are accessible through GEO Series accession number GSE101927.

### Differential gene expression analysis

To identify ancestry-associated differentially expressed genes in Luminal tumors, we categorized patients according to the average European or IA ancestry fractions and compared Luminal B tumors vs. Luminal A tumors. We used Luminal A tumors as a reference group as they represent the most biologically similar but less aggressive breast cancer subtype compared to Luminal B. We used DESeq2 package [34] in R-studio (http://www.rstudio.com/) to perform differential expression analysis. This analysis applies a general linear model to estimate log2 fold changes (log2FC) to test if differences between groups are equal to zero. Pre-filtering was applied to the data matrix to analyze transcripts with at least 1 read count. Genes with Benjamini-Hochberg adjusted < 0.05 (padj < 0.05) were reported as significantly different between groups. Signaling pathway analysis was done in Metacore (Thomson Reuters) and DAVID annotation tool (http://david.abcc.ncifcrf.gov/) [35]. Venn diagrams were done using Venny 2.1 online tool (http://bioinfogp.cnb.csic.es/tools/venny/).

### Real time-PCR validation

cDNA was synthesized from 100ng of total RNA using SuperScript III First-Strand Synthesis SuperMix Kit (Invitrogen) in 166 samples from Luminal tumors (42 analyzed by RNA-seq and 124 new samples), according to the manufacturer’s instructions. TaqMan probes were used to quantify the levels of mRNA expression of candidate genes: *ERBB2* (Hs01001580_m1), *GRB7* (Hs00917999_g1), *ONECUT2* (Hs00191477_m1). The reaction was amplified in a QuantStudio 12 K plex Real-Time PCR machine (ThermoScientific). The 2^-ΔΔCT^ method was used to estimate the fold changes and *GAPDH* (Hs03929097_g1) was used as an internal calibrator. Water was used as a negative control.

### Statistical analysis

All statistical analyses were performed using R project (www.r-project.org) and SPSS Inc. (Released 2007; SPSS for Windows, Version 16.0. Chicago, IL, USA). Differences in the characteristics of the patients according to intrinsic subtype were analyzed using *X*^*2*^ test and differences in the mean of the ancestry fractions, age at diagnosis and tumor size were analyzed using analysis of variance test (ANOVA). *p* values less than 0.05 were considered statistical significant. Logistic regression model was used to test the association between gene expression of *TOP2A* and *CYP19A1* and presence of recurrences.

Pearson correlation was used to determine the correlation between the expression level of ERα, PgR, HER2 and Ki-67 obtained by IHC and RNA-seq. For gene expression, we used normalized values of the read counts from each gene. The expression by IHC was assessed by percentage of expression for ERα, PgR and Ki-67. HER2 measurement was semi-quantitative according to the recommendations of the American Society of Clinical Oncology (ASCO)/College of American Pathologists (CAP) guideline [36]. According to these criteria, negative cases are those with no membrane staining or weak staining for less than 10% of tumor cells (score 0), or incomplete and weak staining for more than 10% of tumor cells (score 1+). Cases with weak to moderate staining in more than 10% of tumor cells are assigned at score of 2+. Finally, HER2 positive cases have a complete and intense membrane staining in more than 10% of tumor cells and are assigned a score of 3+. We used HER2 scores by IHC to performed Pearson correlation.

We used Spearman correlation to test the correlation between the expression levels of the candidate genes obtained by RNA-seq and the fold changes calculated from the qRT-PCR. Linear regression analysis was used to test the association between expression levels of candidate genes and the intrinsic subtypes of breast cancer (Luminal A or Luminal B), and/or the genetic ancestry.

## Results

### Characteristics of patients

Twenty one (21) of these patients were classified as Luminal A and 21 as Luminal B (Table 1) according to the recommendations of the St. Gallen 2013 panel [29].

**Table 1:** Characteristic of patients analyzed by RNA-seq.

	Luminal A (n = 21)	Luminal B (n = 21)	*p*
Age, Yrs.	57.7 ± 13.9	61.1 ± 11.3	0.391
Tumor Size (mm)	78.9 ± 186.6	39.5 ± 20.07	0.871
Mean European ancestry	0.57 ± 0.13	0.58 ± 0.18	0.78
Mean IA ancestry	0.37 ± 0.13	0.36 ± 0.17	0.853
Mean African ancestry	0.06 ± 0.07	0.06 ± 0.06	0.802
**PgR expression, N (%)**			0.11
Positive	21 (100)	17 (81)	
Negative	0	4 (19)	
**HER2 expression, N (%)**			< 0.01
Positive	0	13 (61.9)	
Negative	21 (100)	8 (38.1)	
**Tumor Grade, N (%)**			0.008
I	3 (14.3)	0	
II	16 (76.2)	12 (57.1)	
III	0	6 (28.6)	
Unknown	2 (9.5)	3 (14.3)	
**Nodes, N (%)**			0.354
Positive	9 (42.9)	13 (61.9)	
Negative	12 (57.1)	8 (38.1)	
**AJCC Stage, N (%)**			0.328
I	3 (14.3)	1 (4.8)	
IIA/IIB	9 (42.9)	7 (33.3)	
IIIA/IIIB/IIIC	8 (38.1)	13 (61.9)	
IV	1 (4.8)	0	
**Adjuvant Therapy, N (%)**			0.032
Chemotherapy	1 (4.8)	0	
Hormonotherapy	8 (38.1)	1 (4.8)	
Combined*	11 (52.3)	19 (90.4)	
Not administered	1 (4.8)	0	
Unknown	0	1 (4.8)	
**Cytotoxic regimen, N (%)**			0.003
Anthracyclines	4 (19)	5 (23.8)	
Anthracyclines + Taxanes	2 (9.5)	1 (4.8)	
Anthracyclines + Taxanes + Trastuzumab	0	1 (4.8)	
Anthracyclines + Trastuzumab	0	1 (4.8)	
CMF regimen	0	1 (4.8)	
Taxanes	6 (28.6)	2 (9.5)	
Taxanes + Trastuzumab	0	6 (28.6)	
TC regimen	0	1 (4.8)	
Trastuzumab alone	0	1 (4.8)	
Not administered	9 (42.9)	1 (4.8)	
Unknown	0	1 (4.8)	
**Hormonotherapy, N (%)**			0.001
Anastrazole	2 (9.5)	1 (4.8)	
Letrozole	1 (4.8)	2 (9.5)	
Not administered	2 (9.5)	0	
Unknown	0	1 (4.8)	
Switch Aromatase Inhibitor	1 (4.8)	11 (52.3)	
Tamoxifen	15 (71.4)	6 (28.6)	
**Recurrence, N (%)**			0.439
Systemic	3 (14.3)	4 (19)	
No recurrences	18 (85.7)	16 (76.2)	
Unknown	0	1 (4.8)	

* Hormonotherapy and chemotherapy

The mean age at diagnosis was 59.4 years and the average of the tumor size was 39 millimeters (mm). The average of European, IA and African ancestry fractions were 0.58, 0.36 and 0.06, respectively. We did not find statistical significant differences in the aforementioned characteristics between Luminal intrinsic subtypes. All patients were positive for expression of ER, meanwhile, PgR was positive in all Luminal A tumors and in 81% of Luminal B tumors. Other clinicopathological variables such as node status and the clinical stage at diagnosis, as defined by the American Joint Committee (AJCC) stage, and recurrences did not differ by Luminal intrinsic subtype.

We found statistical significant differences in HER2 expression by IHC (p < 0.01). All Luminal A tumors were negative for HER2 expression while for Luminal B tumors, 61.9% were positive and 38.1% were negative. We also found statistical significant differences in the tumor grade. Tumor grade 3 was found only for Luminal B intrinsic subtype when compared to Luminal A (28.6% vs. 0%, respectively). The administration of adjuvant therapy differed between the two luminal subtypes (p = 0.032). Patients with Luminal A tumors were more likely to receive hormonotherapy than patients with luminal B subtype (38.1% vs 4.8%); while patients with Luminal B tumors were more likely to receive combined therapy compared to luminal A tumors (90.4% vs. 52.3%, respectively). The cytotoxic regimens were also different between the luminal subtypes (p = 0.003). Patients with luminal B subtype more frequently received cytotoxic treatments with anthracyclines (23.8%) and taxanes plus trastuzumab (28.6%) compared to 42.9% of patients with luminal A tumors who did not received cytotoxic chemotherapy. Finally, we also found statistically significant differences in the administration of hormonotherapy (p = 0.001). Patients with luminal A tumors typically received Tamoxifen (71.4%) while patients with luminal B tumors were more likely to switch to an aromatase inhibitor (52.3%).

### Correlation analysis between immunohistochemistry and gene expression levels

As mentioned above, we used IHC surrogates from St. Gallen 2013 consensus to classify breast cancers into intrinsic subtypes. We performed Pearson correlations to determine whether the immunohistochemical expression of ERα, PgR, HER2 and Ki67 was associated with their gene expression profiles. We found statistically significant correlations between IHC and mRNA expression levels measured by RNA-seq for PgR (R^2^ = 0.737, p < 0.01), ERα (R^2^ = 0.505, p = 0.02), Ki67 (R^2^ = 0.629, p < 0.01) and HER2 (R^2^ = 0.485, p = 0.001) (S1A–S1D Fig). These results suggest that the approximation to Luminal subtypes by IHC is reasonable given the high correlation between protein expression and the gene expression of four of the markers used.

### Gene expression profile of Luminal B tumors in Colombian women classified by St Gallen 2013 surrogates

We have previously reported that according to the St. Gallen 2013 panel surrogates, Luminal B is the most common intrinsic subtype of breast cancer in Colombian-Latinas [28]. In order to analyze the underlying molecular profile of Luminal B tumors in our population we compared tumors classified by IHC as Luminal B versus Luminal A. We found 67 differentially expressed genes (padj < 0.05) from which 39 were up-regulated and 28 down-regulated in the Luminal B subtype (Table 2).

**Table 2:** Differentially expressed genes between Luminal B and Luminal A tumors classified by St. Gallen 2013 surrogates.

Up-regulated genes between Luminal B and Luminal A			Down-regulated genes between Luminal B and Luminal A		
Gene	logFC	padj	Gene	logFC	padj
*CDK1*	1.432	0.004	*RALBP1*	-0.443	0.048
*RP11-510N19*.*5*	1.412	0.016	*RNU5B-1*	-0.713	0.029
*AIF1L*	1.400	0.001	*TTC39C*	-0.721	0.042
*CYP19A1*	1.325	0.030	*RCAN3*	-0.760	0.042
*TOP2A*	1.309	0.011	*KDM4B*	-0.762	0.018
*KIF14*	1.287	0.010	*INO80E*	-0.768	0.034
*DSCAM-AS1*	1.275	0.042	*SNORA54*	-0.798	0.024
*LAD1*	1.249	0.040	*STARD13*	-0.845	0.030
*CD24*	1.228	0.024	*HIPK2*	-0.894	0.034
*CENPF*	1.210	0.001	*ZNF213*	-0.913	0.018
*IQGAP3*	1.180	0.021	*ABAT*	-0.922	0.024
*PGAP3*	1.157	0.033	*RP4-734G22*.*3*	-0.980	0.037
*CDC6*	1.148	0.048	*JMJD8*	-1.002	0.023
*SLC4A8*	1.143	0.006	*ELOVL5*	-1.030	0.018
*BCAS1*	1.135	0.024	*TMEM177*	-1.046	0.018
*ORMDL3*	1.135	0.015	*SNHG8*	-1.059	0.018
*CRABP2*	1.123	0.004	*PAIP2B*	-1.062	0.029
*ASPM*	1.119	0.018	*SNORA76C*	-1.095	0.049
*STARD3*	1.113	0.029	*FGD3*	-1.096	0.026
*BUB1*	1.087	0.016	*RPS16P5*	-1.117	0.048
*CCNA2*	1.080	0.034	*FCGBP*	-1.222	0.048
*IGFBP5*	1.062	0.027	*RBBP8*	-1.226	0.013
*MKI67*	1.054	0.021	*KCND3*	-1.228	0.024
*ANLN*	1.048	0.048	*RNU6-36P*	-1.232	0.042
*CCNB2*	1.026	0.043	*BAI2*	-1.372	0.013
*ELF3*	1.018	0.018	*ABCA3*	-1.383	0.001
*EXOC2*	0.982	0.018	*SERPINA1*	-1.449	0.013
*CENPE*	0.976	0.033	*NTRK2*	-1.497	0.008
*NT5E*	0.941	0.014			
*SIX4*	0.918	0.048			
*CLDN4*	0.913	0.038			
*ARF6*	0.810	0.019			
*CDK12*	0.808	0.030			
*RHOC*	0.639	0.049			
*RBM39*	0.568	0.045			
*CAMSAP2*	0.545	0.034			
*UTP20*	0.543	0.043			
*U2SURP*	0.387	0.034			
*CAND1*	0.361	0.034			

**Fig 1:**
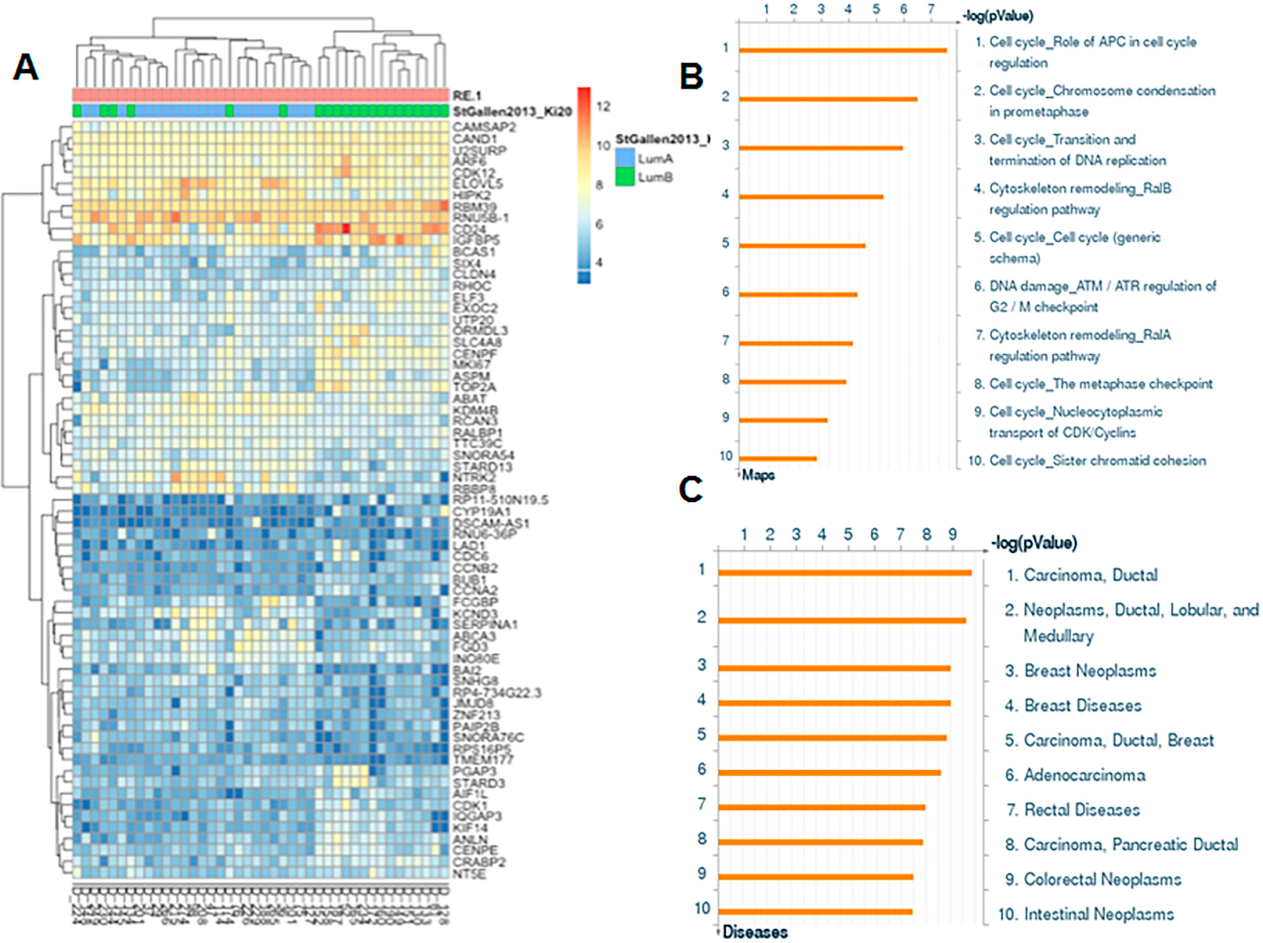
Gene expression profile of 42 Luminal breast cancer samples. (A) Unsupervised hierarchical clustering with 67 differentially expressed genes between IHC defined Luminal B and Luminal A tumors. (B) Most relevant signaling pathways associated with 67 differentially expressed genes in Luminal B tumors from Colombian women. (C) Diseases associated with differentially expressed genes in Luminal B.

Unsupervised hierarchical clustering showed that using these genes, most Luminal B tumors (15) clustered together and diverge from Luminal A tumors (Fig 1A). Interestingly, 6 Luminal B tumors clustered with the Luminal A group. We analyzed the expression levels of *ESR1*, *PGR*, *MKI67* and *ERBB2* at the gene expression level as these codify for the markers that we used in the IHC to distinguish Luminal B from Luminal A tumors following St. Gallen surrogates. We observed that although these 6 tumors have lower expression of *ESR1* when compared to all other luminal A tumors, their expression for the other three markers (PGR, MKI67, and ERBB2) was similar to the luminal A cluster (Table 3). This result suggests that from the molecular profile, these 6 tumors behave more similarly to luminal A tumors than to luminal B although at the protein level they are classified as luminal B. It remains to be determined whether these tumors have better outcomes than the other Luminal B cases. However, Kaplan-Meier analysis did not show any statistically significant difference between the groups (data not shown).

**Table 3:** Expression of *ESR1*, *PGR*, *MKI67* and *ERBB2* in clusters identified in the unsupervised hierarchical clustering.

	*ESR1*	*PGR*	*MKI67*	*ERBB2*
*Luminal A*	9.98 ± 1.25	8.47 ± 2.22	5.73 ± 0.63	203.44 ± 116.68
Luminal A.1*	9.18 ± 1.65	8.04 ± 2.26	5.24 ± 1.32	134.88 ± 65.27
*Luminal B*	9.82 ± 1.65	7.08 ± 1.87	7.10 ± 0.62	789.49 ± 1244.61

*This group corresponds to the tumors classified as luminal B by immunohistochemistry but that clustered together with luminal A tumors

Pathway analysis showed that the top up-regulated genes participate in biological processes such as mitosis and cell cycle regulation (e.g., *CDK1*, *CDC6*, *CCNB2*, *BUB1*, *CENPF*, *ANLN*, *CENPE*, *CCNA2*, *ASPM*, *MKI67*) and down-regulated genes encode mostly phosphoproteins (e.g., *KCND3*, *RALBP1*, *RCAN3*, *ABCA3*, *RBBP8*, *PAIP2B*, *STARD13*, *ELOVL5*, *HIPK2*, *NTRK2*, *KDM4B*, *BAI2*, *FGD3*) (Fig 1B). The diseases associated with these differentially expressed genes include ductal carcinoma and breast neoplasms (Fig 1C), which was consistent with the origin of the tissue specimens.

Interestingly, two of the genes that we found upregulated were *TOP2A* (log2FC = 1.309, padj = 0.011) and *CYP19A1*, which codify for the aromatase gene (log2FC = 1.325, padj = 0.030). As *TOP2A* has been associated with response to anthracycline-based chemotherapy and aromatase inhibitors are widely used for breast cancer treatment, we explored if the expression of these genes and the intrinsic subtype of breast cancer could be associated with the development of recurrences. We did not find any statistical significant association (S1 Table)

### Ancestry-associated differentially expressed genes in Luminal tumors

#### Stratified analysis by European ancestry fraction

**Fig 2:**
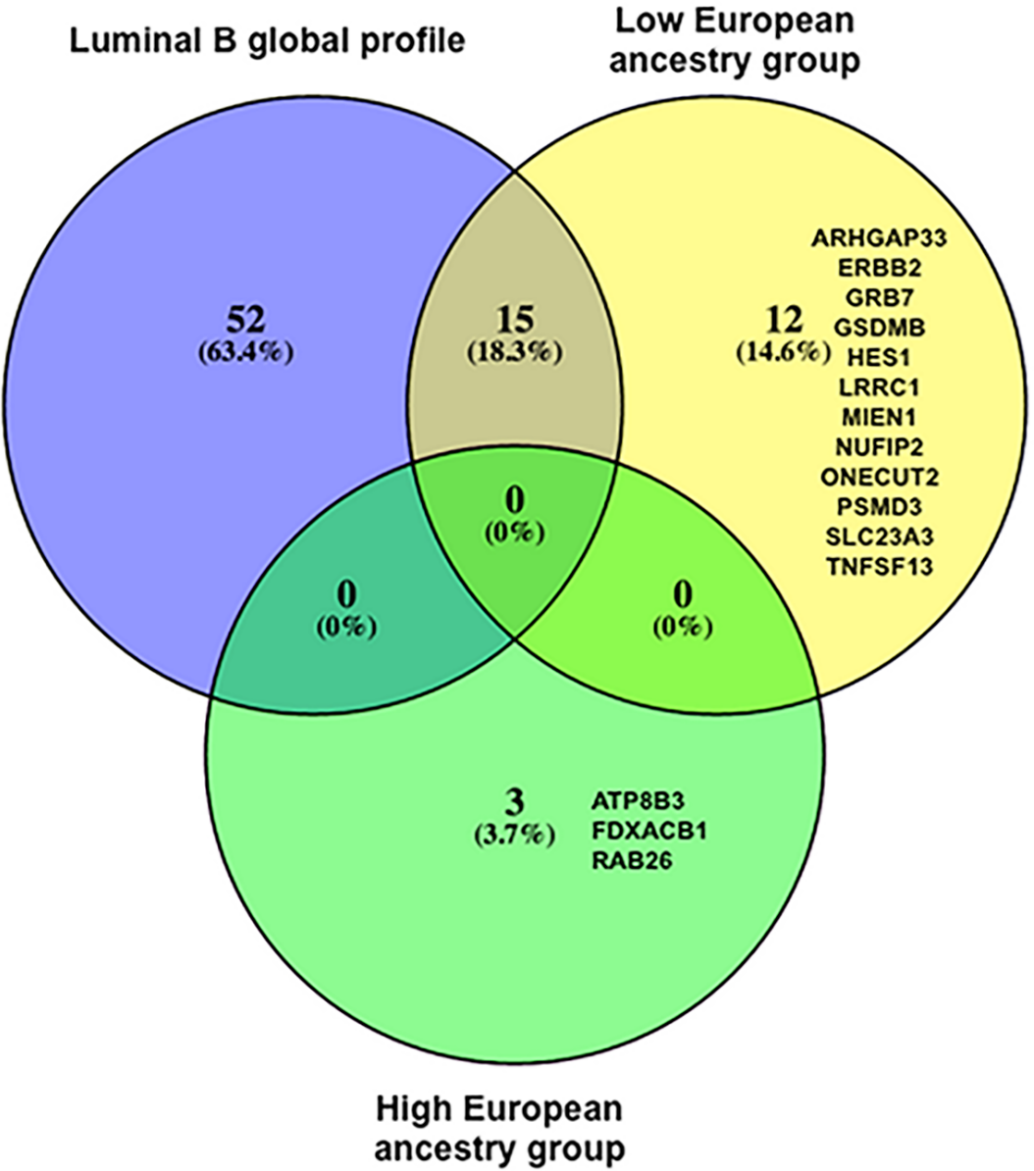
Differentially expressed genes according to European ancestry in IHC defined Luminal B vs. Luminal A tumors. Venn diagram shows the number of differentially expressed genes (padj < 0.05) between Luminal B and Luminal A tumors with low European ancestry and high European ancestry. “Global” refers to differentially expressed genes between Luminal tumors without stratification by ancestry.

To identify ancestry-associated differentially expressed genes in Luminal tumors, we categorized patients according to Luminal subtype (Luminal A and Luminal B) and to the proportion of European ancestry into **low European ancestry group** (European ancestry proportion below the average 0.58); and **high European ancestry group** (European ancestry proportion above the average 0.58). The averages for European, IA and African ancestry fractions according to the assigned groups are shown in the S2 Table. We then compared Luminal B against Luminal A tumors according to the assigned ancestry groups and found 27 ancestry-modulated genes in the low European ancestry group and 3 in the high European ancestry group (Fig 2).

We compared the differentially expressed genes found in the global profile of Luminal B tumors (Table 2) against the ancestry-modulated genes and we observed that 15 genes were in common with the low European ancestry group (*AIF1L*, *CYP19A1*, *CENPF*, *PGAP3*, *SLC4A8*, *ORMDL3*, *CRABP2*, *STARD3*, *BUB1*, *CDK12*, *SNORA54*, *HIPK2*, *FCGBP*, *RBBP8*, *NTRK2*). On the other hand, 12 genes were unique for this ancestry group and included *ERBB2*, *GRB7*, *MIEN1*, *ONECUT2*, *GSDMB*, *NUFIP2*, *TNFSF13*, *LRRC1*, *PSMD3*, *SLC23A3*, *ARHGAP33* and *HES1*. The high European ancestry group did not show common genes with the global profile or with the low European ancestry group and had 3 unique differentially expressed genes (*ATP8B3*, *FDXACB1*, and *RAB26*) (Fig 2 and Table 4).

**Table 4:** Differentially expressed genes for Luminal B vs. Luminal A tumors according to European ancestry group.

Differentially expressed genes unique for Luminal B tumors from the low European ancestry group			Differentially expressed genes unique for Luminal B tumors from the high European ancestry group		
Genes	logFC	padj	Genes	logFC	padj
*ERBB2*	2.367	1.48E-06	*ATP8B3*	-1.70	0.007
*GRB7*	2.327	3.15E-04	*FDXACB1*	-1.62	0.049
*ONECUT2*	2.204	1.28E-03	*RAB26*	-1.72	0.049
*MIEN1*	2.195	3.15E-04			
*GSDMB*	1.723	1.92E-03			
*PSMD3*	1.386	4.33E-02			
*HES1*	1.092	4.69E-02			
*LRRC1*	1.079	4.33E-02			
*NUFIP2*	1.071	6.27E-03			
*ARHGAP33*	-1.263	4.53E-02			
*TNFSF13*	-1.523	3.83E-02			
*SLC23A3*	-1.589	4.33E-02			

#### Stratified analysis by Indigenous American ancestry fraction

**Fig 3:**
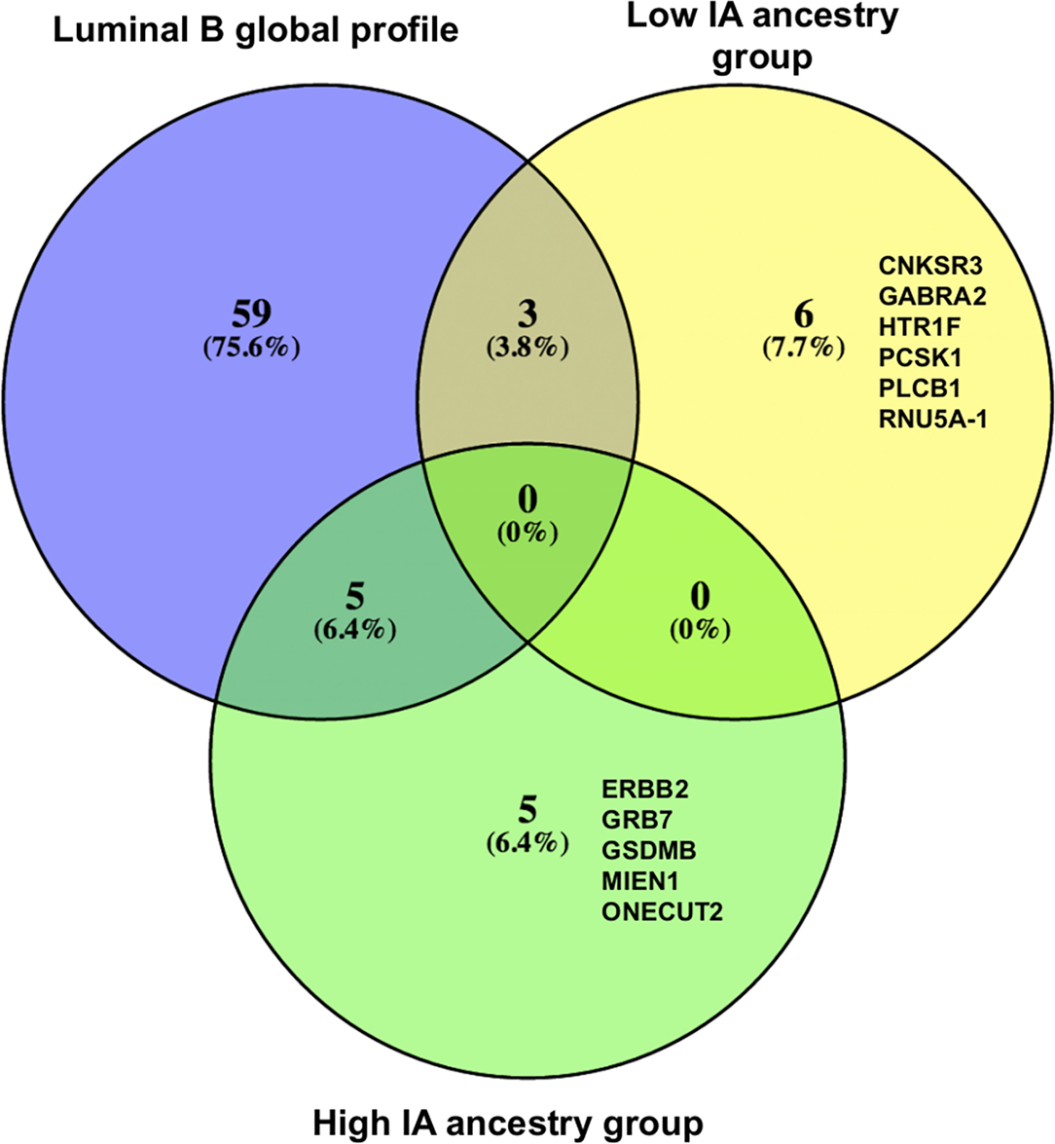
Differentially expressed genes according to IA ancestry in Luminal B vs. Luminal A tumors. Venn diagram shows the number of differentially expressed genes (padj < 0.05) between Luminal B and Luminal A tumors with low IA ancestry and high IA ancestry. “Global” refers to differentially expressed genes between Luminal tumors without stratification by ancestry.

We repeated the differential expression analysis stratifying by the IA average fraction into **low IA ancestry group (**IA ancestry fraction below 0.36), and **high IA ancestry group** (IA ancestry fraction above 0.36). The averages for the European, IA and African ancestry fractions according to assigned groups are in the S3 Table. Compared to the global profile of Luminal B tumors (Table 2), we found 5 genes in common with the high IA ancestry group (*ORMDL3*, *STARD3*, *SLC4A8*, *CDK12*, *HIPK2*) and 3 with the low IA ancestry group (*NT5E*, *SNORA76C*, *ABCA3*) (Fig 3).

In the low IA ancestry group, 6 genes were unique (*PCSK1*, *GABRA2*, *HTR1F*, *CNKSR3*, *PLCB1* y *RNU5A-1*). On the other hand, the unique genes found in the high IA ancestry group were *ERBB2*, *GRB7*, *GSDMB*, *MIEN1* and *ONECUT2*. These 5 genes were also found in the low European Ancestry group and the direction of the change was the same as that of the high IA ancestry group (Table 5).

**Table 5:** Differentially expressed genes in Luminal B tumors according to the Indigenous American ancestry groups.

Differentially expressed genes unique for Luminal B tumors from low IA ancestry group			Differentially expressed genes unique for Luminal B tumors from high IA ancestry group		
Genes	logFC	padj	Genes	logFC	padj
*PCSK1*	-3.34	3.5E-10	*GSDMB*	1.98	0.000
*GABRA2*	-2.27	1.7E-03	*MIEN1*	1.97	0.013
*HTR1F*	-2.23	1.8E-03	*ERBB2*	1.92	0.012
*CNKSR3*	-1.52	7.0E-03	*GRB7*	1.87	0.033
*PLCB1*	1.75	1.4E-02	*ONECUT2*	1.83	0.047
*RNU5A-1*	-1.07	4.0E-02			

### Confirmatory analysis of candidate genes by RT-PCR

We selected *ERBB2*, *GRB7* and *ONECUT2* for validation due to their importance in the biology of breast cancer, the magnitude of the change found in the RNA-seq data analysis and the consistency between the European and IA ancestry analyses. Confirmatory analysis was performed by semi-quantitative RT-PCR in 166 samples from Luminal tumors (42 analyzed by RNA-seq and 124 new samples). The characteristics of the 124 additional patients from the confirmatory analysis are in the S4 Table. Spearman correlation analysis showed statistically significant correlations in gene expression levels between RNA-seq and RT-PCR analysis for *ERBB2* (p < 0.01, R^2^ = 0.62), *ONECUT2* (p = 0.014, R^2^ = 0.62), and *GRB7* (p = 0.0131, R^2^ = 0.40) (S2A–S2C Fig).

We used a linear regression model to test if changes in expression levels of *ERBB2*, *ONECUT2* and *GRB7* were explained by genetic ancestry in Luminal subtypes. For this analysis we used log2FC values from the qRT-PCR. We tested the association of the expression levels of the candidate genes with an interaction variable between intrinsic subtype and genetic ancestry (European or IA). We found a statistically significant association between *ERBB2* expression and the IA fraction (p = 0.02, B = 3.11, CI 95% 0.43, 5.79), but not for the interaction (Table 6).

**Table 6:** Association between candidate genes expression and the interaction between Indigenous American ancestry and intrinsic subtype.

	*ERBB2*				*GRB7*				*ONECUT2*			
	B	p	IC 95%		B	p	IC 95%		B	p	IC 95%	
**IA ancestry fraction**	3.11	0.02	0.43	5.79	0.42	0.80	-2.82	3.65	-2.98	0.47	-11.22	5.26
**Intrinsic subtype**	0.84	0.27	-.065	2.34	-0.75	0.44	-2.68	1.18	-1.87	0.40	-6.38	2.63
**Interaction**	-1.60	0.36	-5.07	1.86	3.12	0.17	-1.37	7.61	6.60	0.18	-3.31	16.52

The association with the European ancestry fraction was not significant (Table 7).

**Table 7:** Association between candidate genes expression and the interaction between European ancestry and intrinsic subtype.

	*ERBB2*				*GRB7*				*ONECUT2*			
	B	p	IC 95%		B	p	IC 95%		B	p	IC 95%	
**European ancestry fraction**	1.00	0.44	-1.56	3.55	-0.08	0.96	-3.16	2.99	-5.69	0.13	-13.11	1.74
**Intrinsic subtype**	0.89	0.32	-0.89	2.68	1.10	0.35	-1.20	3.39	0.37	0.87	-4.09	4.84
**Interaction**	-1.43	0.40	-4.80	1.93	-1.10	0.62	-5.44	3.24	1.91	0.67	-6.99	10.80

**Fig 4:**
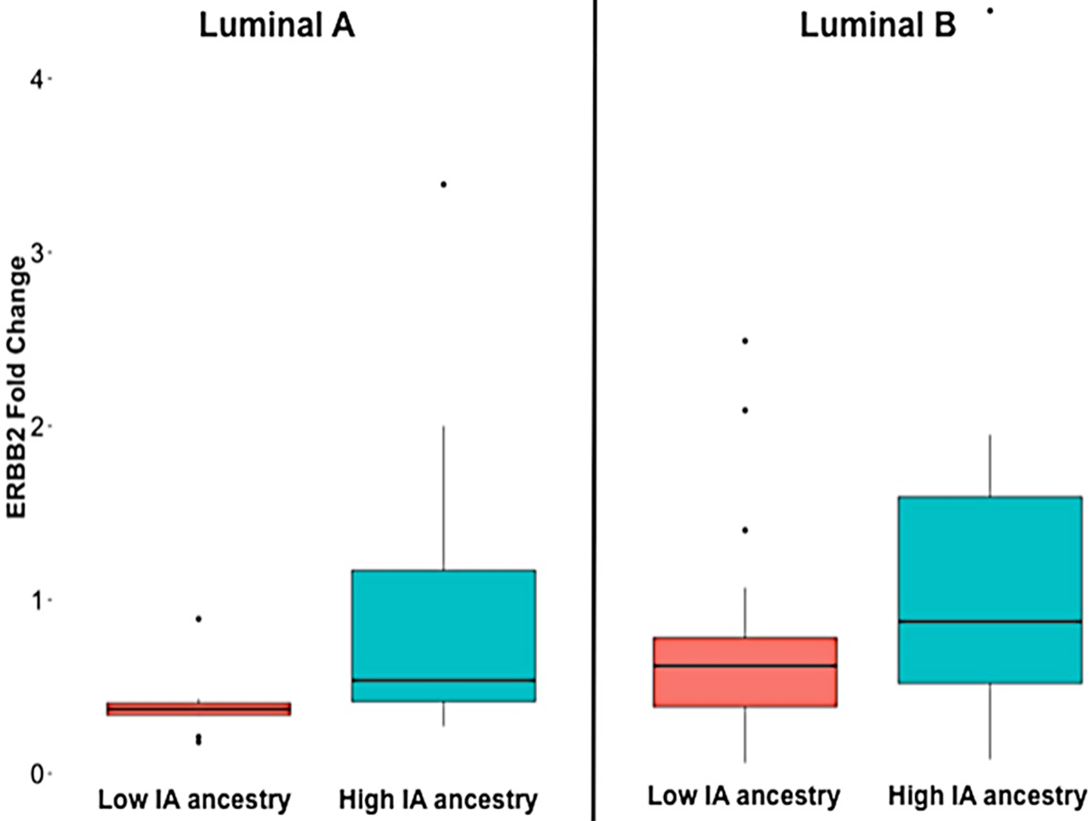
*ERBB2* expression according to intrinsic subtype and Indigenous American ancestry group.

Concordantly, when we tested the association between *ERBB2* expression and the IA ancestry fraction stratified by Luminal subtype, we found an association of *ERBB2* expression and IA ancestry in the Luminal A group (p = 0.009, B = 3.111, CI 95% 0.821, 5.4), and the same trend was observed for the Luminal B group, in which patients with higher IA ancestry showed higher expression of *ERBB2* (Fig 4).

**Fig 5:**
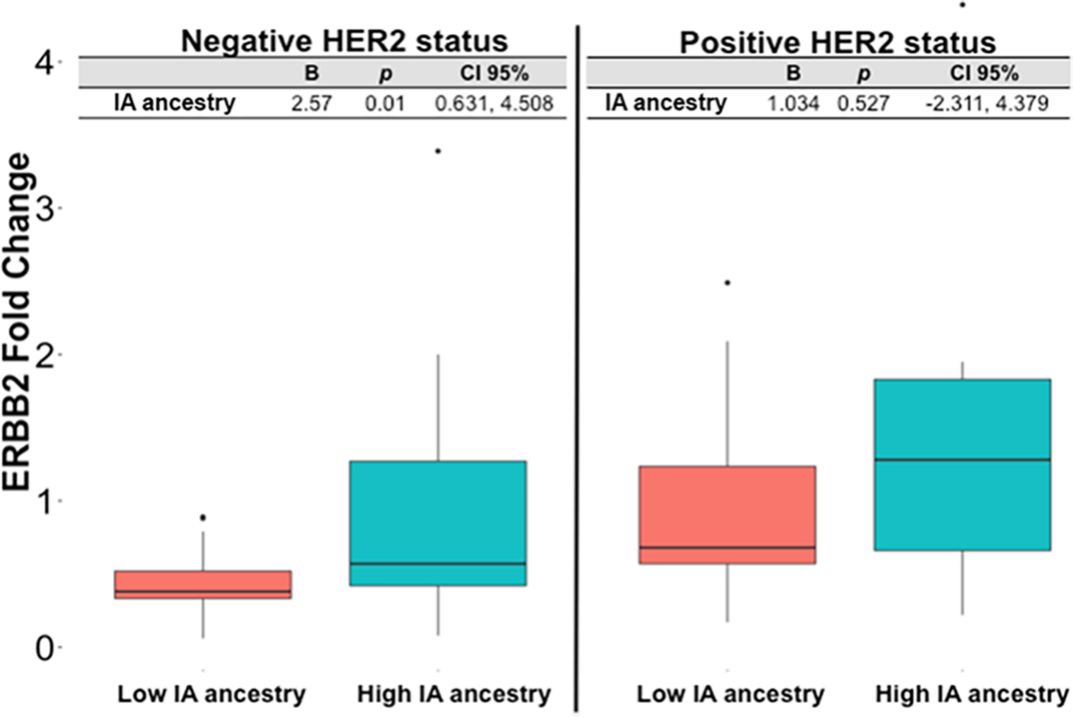
*ERBB2* expression according to HER2 status by immunohistochemistry and Indigenous American ancestry group.

When we conducted the analysis by HER2 status we saw that the association between *ERBB2* expression and ancestry was also independent of immunohistochemical HER2 type (Fig 5).

All together these results suggest that *ERBB2* is a gene positively correlated with IA ancestry in Luminal breast cancer.

## Discussion

Our group has previously shown that Luminal B is the most common intrinsic subtype of breast cancer in Colombian women [28]. Based on that finding we wanted to explore the underlying molecular characteristics of Luminal B tumors in Colombian-Latina women. We found 67 differentially expressed genes between Luminal B and Luminal A tumors. Genes that were up-regulated in luminal B tumors included, *CDK1*, *BUB1*, *CENPF* and *MKI67*, which participate in cell proliferation pathways consistent to what has been reported for the molecular profile in luminal B tumors in other population groups [7, 13, 14, 37–39].

Another up-regulated gene in luminal B tumors was *CYP19A1* that encodes aromatase, the enzyme that catalyzes the rate-limiting step in estrogen biosynthesis, aromatization of androstenedione and testosterone to estrone and estradiol, respectively [40, 41]. Aromatases are highly expressed in breast cancer tissue when compared to normal breast tissue [42] thus it has been suggested that the *CYP19A1* gene participates in the development and progression of breast cancer [41]. Aromatase inhibitors (AIs) that selectively inhibit aromatase activity in peripheral tissues have become a successful therapy for postmenopausal women with hormone-sensitive breast cancer [43, 44]. To the best of our knowledge, this is the first report to show differential expression of *CYP19A1* by luminal intrinsic subtype of breast cancer. The overexpression of this gene in Luminal B tumors in Colombian patients suggests that this subtype may be more sensitive to aromatase inhibitors compared to Luminal A tumors.

DNA topoisomerase IIA (TOP2A) is an isoform of TOP2 enzyme that exerts catalytic activity to induce breaks in double-stranded DNA to release torsional stress. These breaks are subsequently resealed [45]. Sparano et al. [46] suggested that in breast cancer patients with hormone receptor positive and HER2-normal expression, high levels of *TOP2A* could be associated with resistance to antracycline-based chemotherapy. This suggestion came from the finding that higher expression of TOP2A correlated with poor tumor grade and high recurrence score based on the Oncotype Dx signature. Romero et al. [45] found higher expression of TOP2A in Luminal B, HER2-enriched and basal-like when compared to Luminal A subtype, which is consistent with our finding.

As Hispanic/Latinas represent a heterogeneous population group with variation in the European, IA and African ancestry fractions [47], we explored the role of genetic ancestry as a modifier of the molecular characteristics of Luminal tumors in Colombian women. We found 5 genes potentially modulated by genetic ancestry and differentially expressed between Luminal B and Luminal A tumors (*ERBB2*, *GRB7*, *ONECUT2*, *MIEN1* and *GSDMB*). These genes were ancestry-modulated in the analysis based on the European ancestry categories as well as the IA categories.

The *ERBB2*, *GRB7* and *MIEN1* genes, which are located on chromosome 17 in relative proximity within a region including approximately 60,000 base pairs, have been reported co-amplified and associated with poor prognosis in breast cancer [48, 49]. *ERBB2* is located in locus 17q12 and encodes a 185 KDa transmembrane glycoprotein receptor that belongs to the family of the epidermal growth factor receptor (EGFR) [50–52]. It has been reported over-expressed or amplified in 30% of breast tumors and also in ovarian, gastric, and uterine tumors [53]. *GRB7* gene is located in locus 17q11–21 and encodes for an adapter protein present in the cellular cytoplasm and interacts with multiple proteins including tyrosine kinase receptors such as EGFR and ERBB2 (HER2) through its SH2 domains [54, 55]. *GRB7* has been found to regulate migration [56–58] and recently Nadler et al. [54] found that HER2/GRB7 co-expression conferred worse prognosis than *HER2* amplification alone, and that high expression of GRB7 at the protein level is associated with shorter survival times. Finally, *MIEN1*, located in the chromosomal region 17q12-21, was recently discovered [49, 59] and has been associated with enhanced migration in several types of cancer [48, 60]. To the best of our knowledge, this report is the first to show differential expression of *MIEN1* by breast cancer intrinsic subtype and genetic ancestry and to show differential expression of *ERBB2* and *GRB7* by genetic ancestry in breast cancer patients.

After assessing the correlation between RNA-seq and RT-PCR assays, we were able to validate our observation of effect modification of Luminal B vs. Luminal A tumors differentially expressed genes by genetic ancestry for three of the five genes (*ERBB2*, *GRB7 and ONECUT2)*. In the validation set we analyzed a higher number of patients with Luminal tumors (124 additional luminal tumors) and found a significant statistical association between *ERBB2* expression levels and IA ancestry fraction, which seem to be independent of Luminal subtype and immunohistochemical HER2 characterization. This finding suggests that Hispanic/Latina women with higher IA ancestry are more likely to develop Luminal tumors with higher expression of *ERBB2* compared to women with higher European ancestry. However, the relationship between expression of *ERBB2* and HER2 IHC classification needs to be better understood, given that the association between ancestry and *ERBB2* was not paralleled by the association between genetic ancestry and Luminal subtype based on IHC classification. More patients, not only with Luminal tumors but all different subtypes, will be needed to replicate this finding and explore the prognostic value of the association and relevance for the use of trastuzumab treatment in this population. Future studies should confirm if these three genes (*ERBB2*, *GRB7* and *MIEN1*) as they have been reported co-amplified, are also ancestry-modulated together or if the modulation is independent of their co-amplification.

This is the first study, to the best of our knowledge, to explore differences in the molecular profile of an intrinsic subtype of breast cancer according to genetic ancestry in a highly admixed Latin American population. Some studies have compared the molecular profiles of breast cancer between Caucasian and African American women [61–65] in order to seek mechanistic explanations for the differences in disease biology and outcomes observed between these two populations. However, only few studies have included Hispanic/Latina women. Chavez-MacGregor et al. [66] explored differences in the transcriptome and protein expression according to race/ethnicity and intrinsic subtype in 376 women (46 African-American, 47 Hispanic/Latinas and 147 Non- Hispanic white women). They did not find any statistically significant differences in the molecular profiles or at the protein level between the groups they analyzed. However, they did not include genetic ancestry in their analyses, and therefore were unable to assess more subtle differences in expression based on the ancestral genetic architecture of the admixed genomes of African Americans and Hispanic/Latinas.

We are aware of the limitations of our study. First, the approximation of intrinsic subtypes based on IHC and not gene expression could lead to misclassification of tumors. However, when we compared the percent expression of ER/PR/HER2/Ki67 based on IHC and gene expression, we found relatively strong correlations. We are also aware that to find differentially expressed genes by genetic ancestry it would have been more informative to analyze patients with higher variations in their European, IA and African fractions. Nevertheless, by analyzing patients from only one Colombian region (Andean), we aimed at reducing possible differences in expression due to variation in environmental exposures. One significant advantage of the present study was the fact that we analyzed gene expression differences in an admixed group of patients according to their genetic ancestry and not by their self-identification. Finally, it is important to highlight that all the RNA-seq and RT-PCR data shown in this study were obtained from FFPE samples, which confirms their value as a source of information for future work.

## Conclusions

Our results suggest that the expression of *ERBB2*, a crucial gene in breast cancer tumor subtype classification associated with poor prognosis, might be associated with genetic ancestry in breast tumor samples from Colombian women. Women with higher IA ancestry express higher levels of *ERBB2*. Further analyses are necessary to further confirm this association, assess the impact that this association has on HER2 IHC classification, and explore its prognostic value.

## Supporting information

S1 Fig Pearson correlation analysis for expression levels of ER, PR, Ki67 and HER2 assessed by immunohistochemistry and RNA-seq in 42 sequenced patients. X-axis represents the measure by immunohistochemistry and Y-axis measure by RNA-seq. (**A)** Scatter plot from Progesterone receptor expression. (**B)** Scatter plot for estrogen receptor expression. (**C)** Scatter plot for Ki67 expression. (**D**) Scatter Plot for HER2 expression.
(PDF)

S2 Fig Spearman correlation plots show a positive correlation between gene expression values obtained by RNA-seq and RT-PCR. **(A)** Scatter plot for *ERBB2*. **(B)** Scatter plot for *GRB7*. **(C)** Spearman correlation for *ONECUT2*.
(PDF)

S1 Table Association between the expression level of CYP19A1, TOP2A and intrinsic subtype with the presence of recurrences in 42 luminal tumors from Colombian patients. (PDF)

S2 Table Average of different ancestry fractions when patients where stratified according to European ancestry fraction. (PDF)

S3 Table Average ancestry fractions when patients where stratified according to Indigenous American (IA) ancestry fraction. (PDF)

S4 Table Characteristics of the patients analyzed by qRT-PCR included in the validation set. (PDF)
