# Supplementary material for: Ancestry as a potential modifier of gene expression in breast tumors from Colombian women
Source: PLoS One. 2017 Aug 23;12(8):e0183179. doi: 10.1371/journal.pone.0183179 (PMC5568388; doi:10.1371/journal.pone.0183179)
Supplement: S1 Fig — X-axis represents the measure by immunohistochemistry and Y-axis measure by RNA-seq. (A) Scatter plot from Progesterone receptor expression. (B) Scatter plot for estrogen receptor expression. (C) Scatter plot for Ki67 expression. (D) Scatter Plot for HER2 expression. (PDF) [file pone.0183179.s001.pdf]

S1 Fig. Pearson correlation analysis for expression levels of ER, PR, Ki67 and HER2 assessed by immunohistochemistry and RNA-seq in 42 sequenced patients. X-axis represents the measure by immunohistochemistry and Y-axis measure by RNA-seq. (**A)** Scatter plot from Progesterone receptor expression. (**B)** Scatter plot for estrogen receptor expression. (**C)** Scatter plot for Ki67 expression. (**D**) Scatter Plot for HER2 expression.

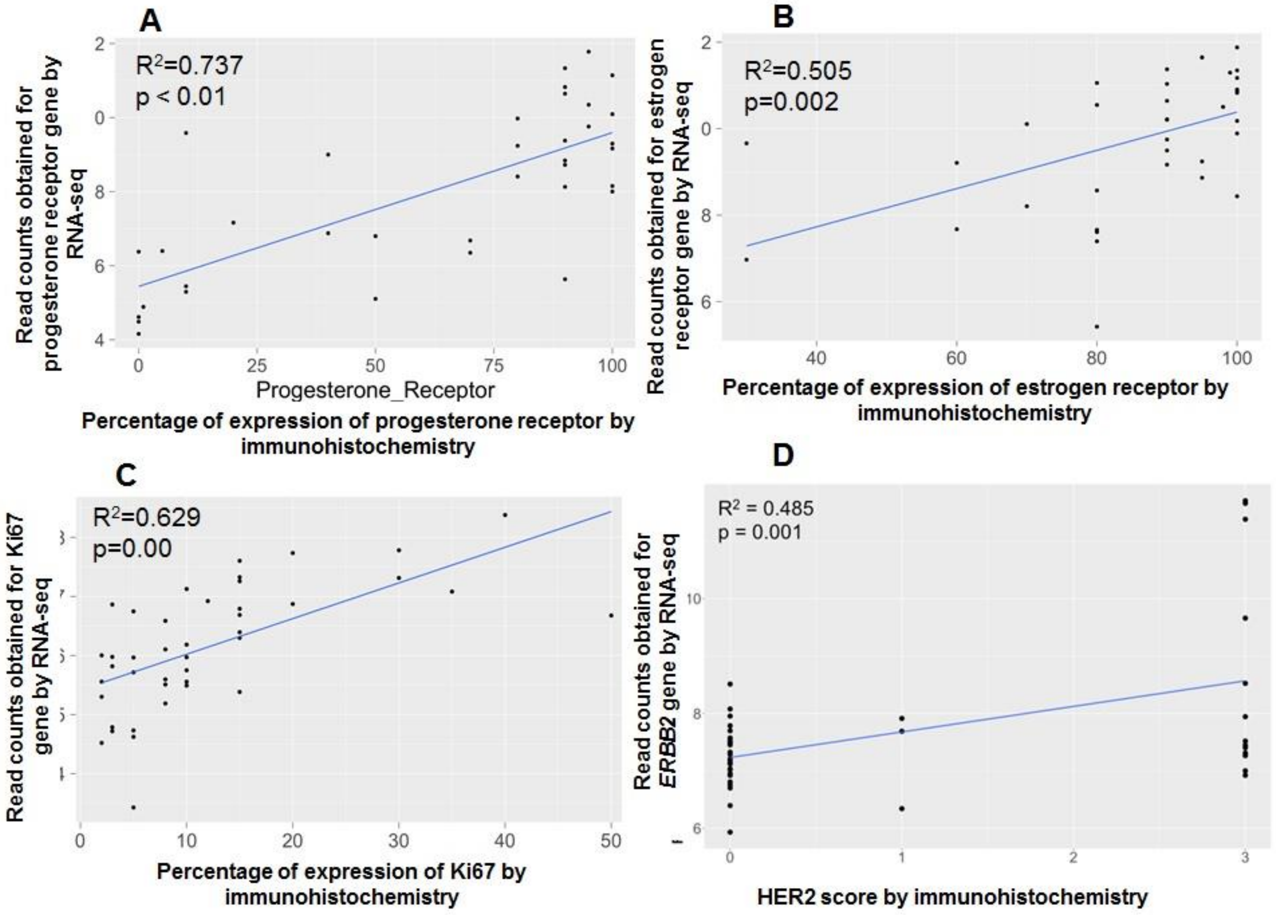

Figure S1
