# Supplementary material for: Ancestry as a potential modifier of gene expression in breast tumors from Colombian women
Source: PLoS One. 2017 Aug 23;12(8):e0183179. doi: 10.1371/journal.pone.0183179 (PMC5568388; doi:10.1371/journal.pone.0183179)
Supplement: S2 Fig — (A) Scatter plot for ERBB2. (B) Scatter plot for GRB7. (C) Spearman correlation for ONECUT2. (PDF) [file pone.0183179.s002.pdf]

S2 Fig. Spearman correlation plots show a positive correlation between gene expression values obtained by RNA-seq and RT-PCR. **(A)** Scatter plot for *ERBB2.* **(B)** Scatter plot for *GRB7.* **(C)** Spearman correlation for *ONECUT2*.

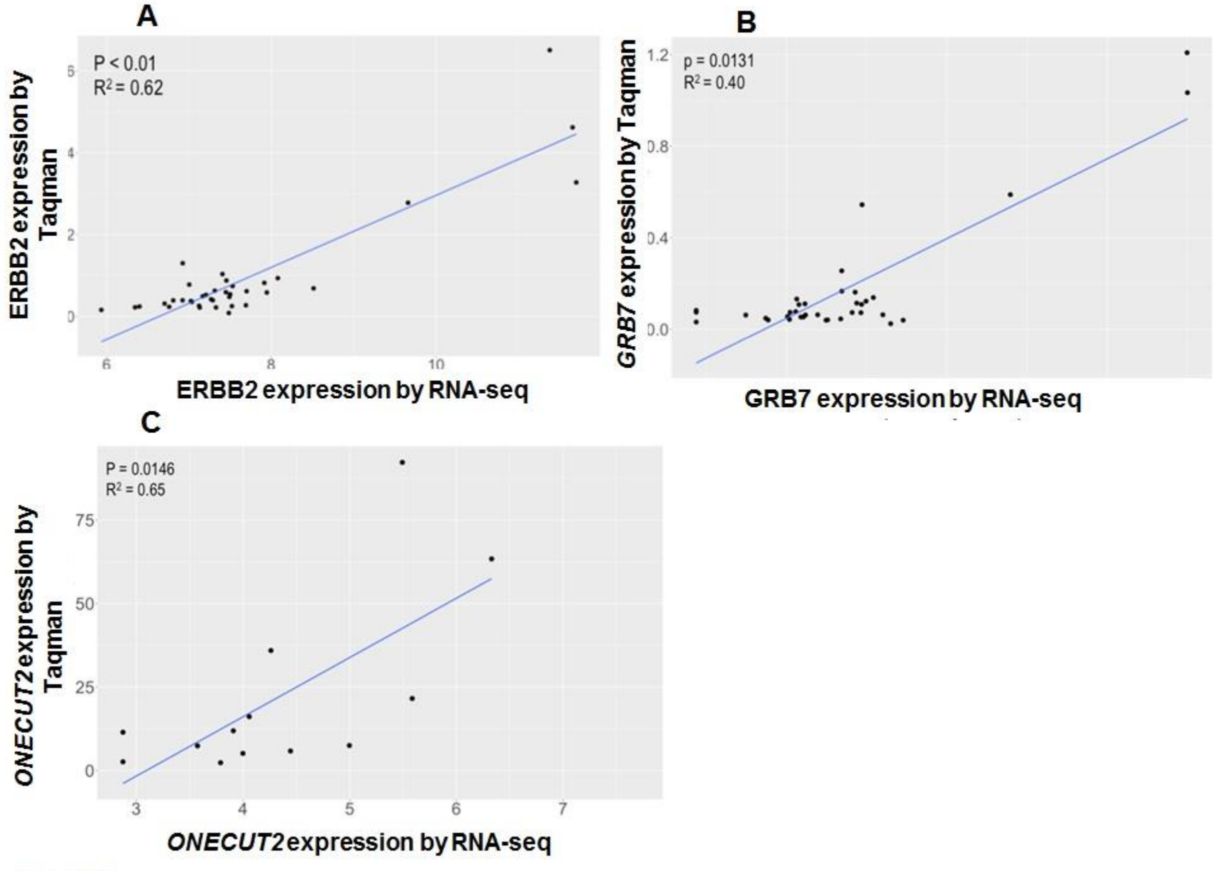

Figure S4
