# Supplementary material for: Ancestry as a potential modifier of gene expression in breast tumors from Colombian women
Source: PLoS One. 2017 Aug 23;12(8):e0183179. doi: 10.1371/journal.pone.0183179 (PMC5568388; doi:10.1371/journal.pone.0183179)
Supplement: S1 Table — (PDF) [file pone.0183179.s003.pdf]

S1 Table. Association between the expression level of *CYP19A1*, *TOP2A* and intrinsic subtype with the presence of recurrences in 42 luminal tumors from Colombian patients

	p	**B**	**95% C.I.**	
CYP19A1	0.261	1.638	0.693	3.873
TOP2A	0.909	0.955	0.434	2.104
**Luminal Subtype**	0.993	1.009	0.138	7.355
