# Supplementary material for: Ancestry as a potential modifier of gene expression in breast tumors from Colombian women
Source: PLoS One. 2017 Aug 23;12(8):e0183179. doi: 10.1371/journal.pone.0183179 (PMC5568388; doi:10.1371/journal.pone.0183179)
Supplement: S2 Table — (PDF) [file pone.0183179.s004.pdf]

S2 Table. Average of different ancestry fractions when patients where stratified according to European ancestry fraction

	**Luminal A (n=21)**		**Luminal B (n=21)**	
	**Low European Ancestry (n=9)**	**High European Ancestry (n=12)**	**Low European Ancestry (n=11)**	**High European Ancestry (n=10)**
Mean European ancestry	0.44 ± 0.07	0.66 ± 0.07	0.44 ± 0.11	0.73 ± 0.08
Mean IA ancestry	0.47 ± 0.05	0.28 ± 0.09	0.48 ± 0.13	0.22 ± 0.06
Mean African ancestry	0.07 ± 0.06	0.05 ± 0.07	0.06 ± 0.05	0.04 ± 0.05
