# Supplementary material for: Ancestry as a potential modifier of gene expression in breast tumors from Colombian women
Source: PLoS One. 2017 Aug 23;12(8):e0183179. doi: 10.1371/journal.pone.0183179 (PMC5568388; doi:10.1371/journal.pone.0183179)
Supplement: S3 Table — (PDF) [file pone.0183179.s005.pdf]

S3 Table. Average ancestry fractions when patients where stratified according to Indigenous American (IA) ancestry fraction.

	Luminal A (n=21)		Luminal B (n=21)	
	**Low IA Ancestry (n=9)**	**High IA Ancestry (n=12)**	**Low IA Ancestry (n=13)**	**High IA Ancestry (n=8)**
Mean European ancestry	0.68 ± 0.07	0.48 ± 0.1	0.69 ± 0.07	0.41 ± 0.1
Mean IA ancestry	0.25 ± 0.08	0.45 ± 0.06	0.24 ± 0.11	0.54 ± 0.09
Mean African ancestry	0.06 ± 0.08	0.06 ± 0.06	0.06 ± 0.06	0.04 ± 0.04
