# Supplementary material for: Ancestry as a potential modifier of gene expression in breast tumors from Colombian women
Source: PLoS One. 2017 Aug 23;12(8):e0183179. doi: 10.1371/journal.pone.0183179 (PMC5568388; doi:10.1371/journal.pone.0183179)
Supplement: S4 Table — (PDF) [file pone.0183179.s006.pdf]

S4 Table. Characteristics of the patients analyzed by qRT-PCR included in the validation set

	**Luminal A (n = 47)**	**Luminal B (n = 77)**	***p***
	**Mean ± SD**	**Mean ± SD**	
Age	58.5 ± 11.4	58.4 ± 11.5	0.957
Tumor Size (mm)	38.6 ± 31.6	41.2 ± 21.1	0.606
Mean European ancestry	0.5 ± 0.16	0.53 ± 0.14	0.243
Mean IA ancestry	0.41 ± 0.14	0.41 ± 0.13	0.897
Mean African ancestry	0.09 ± 0.18	0.06 ±0.07	0.207
	**N (%)**	**N (%)**	
**PgR expression**			< 0.01
Positive	47 (100)	56 (72.7)	
Negative	0	21 (27.3)	
**HER2 expression**			< 0.01
Positive	0	28 (22.6)	
Negative	47 (100)	96 (77.4)	
**Tumor Grade**			0.087
I	3 (6.4)	7 (9.1)	
II	34 (72.3)	47 (61)	
III	4 (8.5)	19 (24.7)	
Unknown	6 (12.8)	4 (5.2)	
**Nodes**			
Positive	23 (48.9)	38 (49.4)	1
Negative	24 (51.1)	37 (48.1)	
Unknown	0	2 (2.6)	
**AJCC Stage**			0.5117
I	2 (4.3)	6 (7.8)	

IIA/IIB	26 (55.3)	31 (27.3)
IIIA/IIIB/IIIC	17 (36.2)	36 (46.8)
IV	2 (4.3)	2 (2.6)
Unknown	0	2 (2.6)
